# Living in uncertain times: trajectories to death in residential care homes

**DOI:** 10.3399/bjgp14X681397

**Published:** 2014-09-01

**Authors:** Stephen Barclay, Katherine Froggatt, Clare Crang, Elspeth Mathie, Melanie Handley, Steve Iliffe, Jill Manthorpe, Heather Gage, Claire Goodman

**Affiliations:** Primary Care Unit, Department of Public Health and Primary Care, University of Cambridge, Cambridge.; International Observatory on End of Life Care, Faculty of Health and Medicine, Lancaster University, Lancaster.; Primary Care Unit, Department of Public Health and Primary Care, University of Cambridge, Cambridge.; Centre for Research in Primary and Community Care, University of Hertfordshire, Hatfield.; Centre for Research in Primary and Community Care, University of Hertfordshire, Hatfield.; Department of Primary Care and Population Health, University College London, London.; Social Care Workforce Research Unit, King’s College London, London.; Department of Economics, University of Surrey, Guildford.; Centre for Research in Primary and Community Care, University of Hertfordshire, Hatfield.

**Keywords:** care homes, dying trajectories, end-of-life care, palliative care, primary health care

## Abstract

**Background:**

Older people living in care homes often have limited life expectancy. Practitioners and policymakers are increasingly questioning the appropriateness of many acute hospital admissions and the quality of end-of-life care provided in care homes.

**Aim:**

To describe care home residents’ trajectories to death and care provision in their final weeks of life.

**Design and setting:**

Prospective study of residents in six residential care homes in three sociodemographically varied English localities: Hertfordshire, Essex, and Cambridgeshire.

**Method:**

Case note reviews and interviews with residents, care home staff, and healthcare professionals.

**Results:**

Twenty-three out of 121 recruited residents died during the study period. Four trajectories to death were identified: ‘anticipated dying’ with an identifiable end-of-life care period and death in the care home (*n* = 9); ‘unexpected dying’ with death in the care home that was not anticipated and often sudden (*n* = 3); ‘uncertain dying’ with a period of diagnostic uncertainty or difficult symptom management leading to hospital admission and inpatient death (*n* = 7); and ‘unpredictable dying’ with an unexpected event leading to hospital admission and inpatient death (*n* = 4). End-of-life care tools were rarely used. Most residents who had had one or more acute hospital admission were still alive at the end of the study.

**Conclusion:**

For some care home residents there was an identifiable period when they were approaching the end-of-life and planned care was put in place. For others, death came unexpectedly or during a period of considerable uncertainty, with care largely unplanned and reactive to events.

## INTRODUCTION

In the UK, 458 000 people live in care home settings^1^ in which 103 000 deaths occurred in England and Wales in 2012 (20.7% of all deaths),^2^ half of such deaths being from dementia.^3^,^4^ Care homes may be categorised into residential care homes that provide personal care only and nursing homes that provide personal and nursing care.^5^,^6^ They are increasingly recognised as important providers of palliative care for older people, potentially offering a homelike environment, continuity of care, and relationship-centred care until death.

Health and social care policy increasingly aspires to high-quality services in all settings.^7^,^8^ UK health policy uses the term ‘end-of-life care’ to refer to care provided in the last year of life. In other countries this may be referred to as ‘palliative care’. Since 2004, the UK end-of-life care service improvement programme has paid specific attention to care homes, advocating use of modified mainstream ‘tools’ such as the Gold Standards Framework,^9^,^10^ Liverpool Care Pathway (LCP),^11^,^12^ Preferred Priorities for Care,^13^ and Advance Care Planning;^14^ frameworks designed to improve team working and communication, with place of death as a key indicator of success. Current policy has an emphasis on facilitating home death,^15^,^16^ and is a source of much debate,^17^ as long ago as 1978, Colin Murray-Parkes pointed out in this Journal that ‘*home can be the best place or the worst place to die*'.^18^ The recent withdrawal of the LCP^19^ and the new guidance issued by the Leadership Alliance for the Care of Dying People^20^ has highlighted the importance of recognising the approach of the end of life where possible, with open communication with patients and families concerning the inherent uncertainties involved.

‘Dying trajectories’ that map recognisable patterns of functional decline over the months prior to death are prominent in discussions of end-of-life care provision,^21^,^22^ especially in relation to people with conditions other than cancer, who are under-represented on GP end-of-life care registers.^23^ While the gradual deterioration or ‘dwindling’ of frail older people is recognised, this is commonly only in retrospect. Little is known about the process of care or how healthcare practitioners work with care homes to support this population.^24^ There is a lack of mapping of events for care home residents, who often experience physical and cognitive frailty, as they approach the end of their lives, to inform the care and support provided by care home staff and primary care teams.

**How this fits in**

Death is often unpredictable among care home residents, occurring in the context of chronic illnesses and prognostic uncertainty. Uncertainty over residents’ proximity to death is difficult for care home staff and primary care clinicians to manage, especially out of hours. End-of-life care tools that aim to optimise care and prevent hospital admissions that may be inappropriate are especially useful when death is anticipated. Tools and support for staff to support older people dying unexpectedly or amidst diagnostic and prognostic uncertainty are still required. A greater understanding of the trajectories to death that older people may experience in care homes should facilitate effective planning and preparation for individual residents, relatives, care homes, and supporting primary care professionals.

This study set out to describe residential care home residents’ trajectories to death and care provision in their final weeks of life.

## METHOD

A prospective study was undertaken for the period 2008 to 2009 using mixed data collection methods that have been described in detail elsewhere.^25^,^26^ The research team approached six residential care homes in three primary care trust areas of England that were of moderate size (30–60 residents) and had recently obtained favourable reports from the Commission for Social Care Inspection (now the Care Quality Commission). Sampling was purposive to include a diversity of providers (charitable, large commercial, and individual private owner) and one home was Gold Standards Framework accredited. Within each care home, residents were approached to give consent for researchers to access their care home and medical records, and to participate in semi-structured interviews. The consultees of those residents who were deemed to lack capacity to give such consent were approached, seeking their opinion as to whether their relative would have been happy to participate and agree to their records being accessed should they have been able to consent. Consent was obtained for 121 residents. Care home staff were asked for their opinions on the care of those who had died.

**Figure 1.**
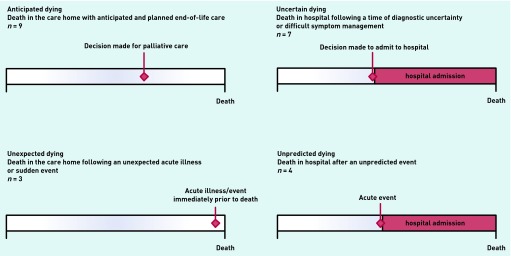
***Trajectories to death in residential care homes during the last month of life.***

This study focuses mainly on the 23 participants who died during the 12 months of data collection (there was a delay in home recruitment that limited data collection at two settings to 6 months only). Data were extracted from their care home records and the small number of GP notes kept in the homes, focusing on changes in residents’ medical condition and mobility, consultations with medical or nursing staff, conversations about future care preferences, and admissions to hospital. These key events during residents’ last 30 days of life were entered into Microsoft^®^ Visio software that has been used in other palliative care studies,^27^ with healthcare professional activity being displayed separately from care home staff comments and observations. Data analysis employed the qualitative approach of framework analysis.^28^ Given the current policy and clinical focus on place of death and identification of those close to death, the dataset was examined with those two themes in mind from the outset. Individual plots of timelines of events were initially examined by two clinically qualified members of the research team, a nurse and a GP, and then discussed more widely within the research team. A typology of four ‘trajectories to death’ was developed from the data, according to whether death occurred in the care home or hospital, and the degree to which the death had been predicted (Figure 1). The 23 deceased participants were then categorised independently by the two researchers, with any disagreements resolved in discussion.

## RESULTS

Nine deaths occurred in the care homes after a period of planned end-of-life care: ‘anticipated dying’. Three deaths occurred in the care homes following an unexpected acute illness or sudden event: ‘unexpected dying’. Seven deaths occurred in hospital after a period of diagnostic uncertainty or difficult symptom management that had led to hospital admission: ‘uncertain dying’. Four deaths occurred in hospital after an unexpected acute event in the care home that had precipitated hospital admission: ‘unpredictable dying’.

### Anticipated dying

Records analysis for the nine residents in the ‘anticipated dying’ category indicated that they were recognised as approaching the end of their lives some time before death, with their dying phase and death managed in the care home. There was documentation of progressive physical deterioration, a focus on ‘tender loving care’, commencement of the LCP, or setting up a syringe driver for subcutaneous drug administration. Three of these residents had cancer, three lived with dementia, and all died in the care home. Pain was recorded as a symptom for six people, and over a prolonged period of time for three individuals.

**Figure 2.**
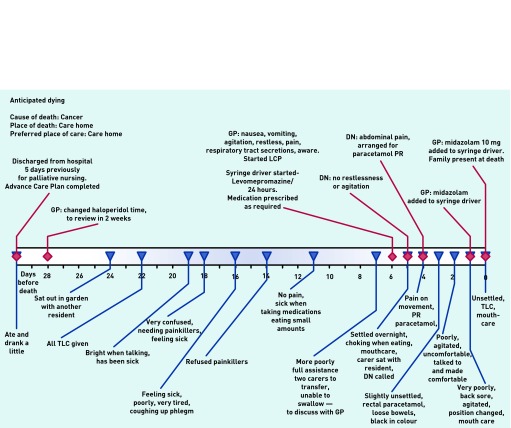
***Patient in ‘anticipated dying’ category. DN = district nurse. TLC = tender loving care.***

Figure 2 illustrates one example of a patient whose death was anticipated. The resident was discharged from hospital with advanced cancer 33 days before death and was monitored by care home staff who involved the GP and district nurses increasingly as death drew near. This resident died peacefully in the care home, on the LCP, with pain-relieving and other medication delivered via a syringe driver and with the family present. Staff reported that they felt the death ‘had gone well’.

### Unexpected dying

**Figure 3.**
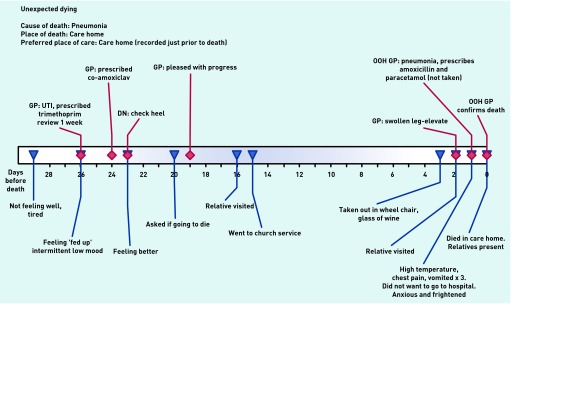
***Patient in ‘unexpected dying’ category. DN = district nurse. OOH = out of hours. UTI = urinary tract infection***

‘Unexpected dying’ was the trajectory for three residents who had been stable and relatively well until an illness arose, that was not initially obviously life-threatening, but which led to death in the care home within a few days. Figure 3 shows one example: a resident’s initial urine infection was treated successfully, but 3 weeks later a chest infection led to an out-of-hours GP being called who respected the resident’s wish not to go to hospital. Care was provided in the home and death occurred 2 days later, with relatives present. The care home staff felt supported by the GP and were pleased that the resident died in their place of choice.

### Uncertain dying

‘Uncertain dying’ was the trajectory for seven residents and was the most complex trajectory. The period before death was a time of diagnostic and prognostic uncertainty, with the residents being unwell but not clearly close to death. They were admitted to hospital for further investigations or for treatment of infections that were not responding to oral antibiotics.

**Figure 4.**
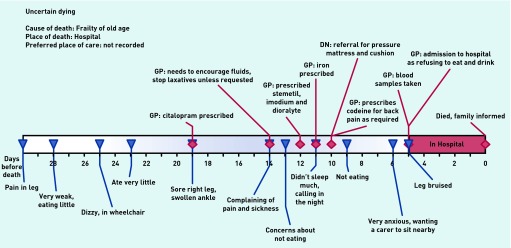
***Patient in ‘uncertain dying’ category.***

Figure 4 outlines one example of a resident who had multiple symptoms which were difficult to manage in the care home: dizziness, vomiting, pain, sleeplessness, problems with skin integrity, and anxiety, with seven GP visits and one district nurse visit during the month prior to admission.

The care home manager’s assessment was that hospital admission had been needed, although she had wanted the resident to return to the care home to die if active treatment was not appropriate. She was disappointed that this did not happen and that the resident had died in hospital.

### Unpredictable dying

‘Unpredictable dying’ involved four residents whose condition had been stable but who suffered an unexpected acute and lethal event, such as a stroke, heart attack or hip fracture, which precipitated admission to hospital, where they later died. These deaths were a challenge to the care home staff, especially if very sudden. Three admissions were via emergency ambulance, and the fourth was after a GP assessment.

**Figure 5.**
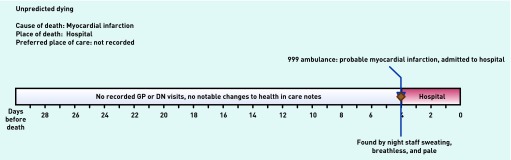
***Patient in ‘unpredicted dying’ category.***

Figure 5 shows an example of a resident in this category. Care home records documented no significant change during the month preceding the resident’s death, but when the resident was found by night staff to be breathless, pale, and sweaty, an emergency ambulance was called. In hospital acute myocardial infarction was diagnosed and the resident died in hospital 4 days later. The care home staff found this death ‘difficult’ as it was unexpected and the resident was particularly popular.

### End-of-life care tools

End-of-life care tools were used infrequently. Only one resident had a Preferred Priorities for Care document, although for seven there was evidence of advance care planning, with their care preferences documented more informally in their care home records. Only one of the 12 residents who died in a care home was on the LCP prior to death. It is unknown how many of the 11 residents who died in hospital were on the LCP prior to death because their hospital records were not accessed.

### Hospital admissions

Urgent transfer to hospital did not usually presage the end of life. Information about hospital admissions was collected for 12 months in four care homes and for 6 months in two. Of the 23 residents who died, eight had contact with either an out-of-hours doctor or ambulance paramedic in the month prior to death: five at the point of death and three resulting in hospital admission. Of the 29 other residents for whom one urgent hospital admission was documented during the study period, 17 were still alive and in the care home at the end of the study period, eight had died, and four had left the care home and were lost to follow-up. Of the 13 other residents who had had two or more urgent hospital admissions during the period documented, six were still alive and in the care home, five had died, and two had left the care home and were lost to follow-up.

## DISCUSSION

### Summary

This study highlights the challenges of providing end-of-life care in care homes with no on-site nursing and which rely mainly on primary care services for medical and nursing care. Four distinct but potentially overlapping trajectories to death were identified. The largest group of dying residents experienced ‘anticipated dying’, with planned provision of end-of-life care in the care home. Others experienced ‘unexpected dying’, where death occurred in the care home after sudden and unexpected events. Others experienced ‘uncertain dying’ where decisions were made to admit them to hospital in the context of clinical and diagnostic uncertainty or failure of initial treatment. A final group experienced ‘unpredictable dying’ in hospital after unexpected events such as a heart attack or hip fracture. Most emergency hospital admissions during the study did not end in death.

While some deaths of care home residents can be anticipated, there are many others where there will be great uncertainty concerning the potential for recovery or death. Care home staff, attending GPs, and ambulance staff making difficult decisions in uncertain circumstances, particularly out of hours, may benefit from considering which of the four identified trajectories the patient or resident may be on, although for some this may only be identified retrospectively.

### Strengths and limitations

This study is the largest study of end-of-life care in residential care homes to date, drawn from a diverse group of six care homes. The prospective and longitudinal nature of the research yielded richer data than has been obtained from the predominantly cross-sectional research undertaken to date: events were documented as they were happening, giving data more grounded in the realities and uncertainties of care in this setting. The data obtained are largely restricted to care home records, which were at times very sparse. It is acknowledged that further significant events such as visits from clinicians and conversations about future care may have taken place but were not documented.

### Comparison with existing literature

The literature concerning dying trajectories has focused on functional decline as death approaches,^21^,^22^ with recent expansion including social, psychological, and spiritual domains.^29^ Most care home residents may be described as already being on the chronic frailty trajectory described earlier.^21^,^22^ The trajectories to death described in this study may be seen as frailty subcategories according to whether death occurred in the care home or the hospital, and the degree to which the death had been anticipated.

While all care home residents may be considered as broadly approaching the end of their lives, the life expectancy of those in residential homes (as in this study) is considerably longer than of nursing home residents. Awareness and open discussion of the unexpected, uncertain, and unpredictable nature of the final weeks and days of life for many residents is of great importance for clinical practice. Conversations with residents, their family members, and care home staff concerning their wishes and preferences for the final days of life may enable the formulation of advance care plans, which have been shown to increase patient satisfaction,^30^ enhance realistic hope,^31^ lead to increased quality of life,^32^ and reduce the time spent in hospital and hospital costs.^33^–^35^

However, it is of note that many of the residents in this study did not wish to have conversations about their wishes for end-of-life care.^25^ This places a greater responsibility on all involved, especially GPs, to ensure that while all care home residents are offered the opportunity to discuss and plan for end-of-life care, these conversations are not imposed.^36^ The completion of an advance care plan does not preclude the potential for individuals to change their minds,^37^ or the need for clinical decision making at the time.^38^ In this study, most emergency hospital admissions were not terminal events.

Many current NHS policies, resources, and tools are predicated on an assumption that it is possible to identify when someone is close to death. This study suggests that while their application may be appropriate for some care home residents, they are not appropriate for all. A professionally driven tick-box approach is to be avoided if care is to be genuinely patient- or resident-centred.^36^ Residents, family members, NHS practitioners, and care home staff all need to accept that for some residents the trajectory to death will be marked by uncertainty, unpredictability, and ambiguity as to the proximity of death.

### Implications for research and practice

Care home residents would all benefit from continuity of GP care: in many practices one GP covers each home and is familiar with each resident’s medical history and wishes, the views of their relatives and staff, and has over a period of time the opportunity to develop an awareness of their illness trajectory. For some with uncertain trajectories, marked by diagnostic uncertainty and challenging symptom control, community geriatricians or palliative care specialists could enable resolution of issues without hospital admission. However, there were many for whom hospital admission immediately prior to death would appear to have been unavoidable and appropriate, given the high proportion of emergency admissions that did not end in death.

This typology of dying trajectories among care home residents would benefit from further study in other settings, particularly in nursing homes. Additional work is also needed to address how health and social care staff can work together to optimally support older people who are in the last period of their lives but not actively dying.^39^

## References

[1] Laing Buisson. Care of Elderly People UK 2012/13 - Key facts:.

[2] (2013). Office for National Statistics. Mortality Statistics 2012.

[3] (2008). National Audit Office. End of Life Care.

[4] Goodman C, Evans C, Wilcock J (2010). End of life care for community dwelling older people with dementia: an integrated review. Int J Geriatr Psychiatry.

[5] Fleming J, Zhao J, Farquhar M (2010). Place of death for the ‘oldest old’: ≥85-year-olds in the CC75C population-based cohort. Br J Gen Pract.

[6] Froggatt K, Davies S, Meyer J (2009). Understanding care homes: a research and development perspective.

[7] Department of Health (2008). End of life care strategy Promoting high quality care for all adults at the end of life.

[8] (2010). Social Care Advisory Group of the National End of life care Programme.. Supporting people to live and die well: a framework for social care at the end of life..

[9] Thomas K (2003). Caring for the dying at home.

[10] Thomas K (2003). The Gold Standards Framework in community palliative care. Eur J Palliat Care.

[11] Ellershaw J, Foster A, Murphy D (1997). Developing an integrated care pathway for the dying patient. Eur J Palliat Care.

[12] Ellershaw J, Wilkinson S (2003). Care of the dying: a pathway to excellence.

[13] Storey L, Pemberton C, Howard A, O'Donnell L (2003). Place of death: Hobson's Choice or patient choice?. Cancer Nurs Pract.

[14] NHS End-of-life care Programme. (2008). Advance care planning: a guide for health and social care staff.

[15] National Institute for Health and Care Excellence. Quality, Innovation, Productivity and Prevention (QIPP) Quality assured examples of improvements in quality and productivity across the NHS and social care.

[16] NHS Scotland (2008). Living and dying well: a national action plan for palliative and end of life care in Scotland.

[17] Barclay S, Arthur A (2007). Place of death: how much does it matter? The priority is to improve end-of-life care in all settings. Br J Gen Pract.

[18] Parkes CM (1978). Home or hospital? Terminal care as seen by surviving spouses. J R Coll Gen Pract.

[19] Knights D, Wood D, Barclay S (2013). The Liverpool Care Pathway for the dying: what went wrong?. Br J Gen Pract.

[20] Leadership Alliance for the Care of Dying People. Once chance to get it right Improving people's experience of care in the last few days and hours of life.

[21] Lunney JR, Lynn J, Foley DJ (2003). Patterns of functional decline at the end of life. JAMA.

[22] Murray SA, Kendall M, Boyd K, Sheikh A (2005). Illness trajectories and palliative care. BMJ.

[23] Omega: the National Association for End of life care. End of life care in primary care: 2009 national snapshot.. http://www.goldstandardsframework.org.uk/cd-content/uploads/files/Library,%20Tools%20%26%20resources/EOLC%20in%20Primary%20Care%20national%20snapshot%20-%20Key%20Findings.pdf.

[24] Nicholson C, Meyer J, Flatley M (2012). Living on the margin: understanding the experience of living and dying with frailty in old age. Soc Sci Med.

[25] Mathie E, Goodman C, Crang C (2012). An uncertain future: the unchanging views of care home residents about living and dying. Palliat Med.

[26] Goodman C, Mathie E, Cowe M (2011). Talking about living and dying with the oldest old: public involvement in a study on end of life care in care homes.. BMC Palliat Care.

[27] Momen N, Kendall M, Barclay S, Murray S (2013). Using timelines to depict patient journeys: a development for research methods and clinical care review. Prim Health Care Res Dev.

[28] Ritchie J, Spencer L, O'Connor W, Ritchie J, Lewis J (2003). Carrying out qualitative analysis. Qualitative research practice: a guide for social science students and researchers.

[29] Murray SA, Kendal M, Grant E (2007). Patterns of social, psychological, and spiritual decline toward the end of life in lung cancer and heart failure. J Pain Symptom Manage.

[30] Detering KM, Hancock AD, Reade MC, Silvester W (2010). The impact of advance care planning on end-of-life care in elderly patients: randomised controlled trial. BMJ.

[31] Davison SN, Simpson C (2006). Hope and advance care planning in patients with end stage renal disease: qualitative interview study. BMJ.

[32] Wright AA, Zhang B, Ray A (2008). Associations between end-of-life discussions, patient mental health, medical care near death, and caregiver bereavement adjustment. JAMA.

[33] Abel J, Pring A, Rich A (2013). The impact of advance care planning of place of death, a hospice retrospective cohort study. BMJ Support Palliat Care.

[34] Meeussen K, Van den Block L, Bossuyt N (2009). GPs' awareness of patients' preference for place of death. Br J Gen Pract.

[35] Badger F, Clifford C, Hewison A, Thomas K (2009). An evaluation of the implementation of a programme to improve end-of-life care in nursing homes. Palliat Med.

[36] Barclay S, Case-Upton S (2009). Knowing patients' preferences for place of death: how possible or desirable?. Br J Gen Pract.

[37] Cosgriff J, Pisani M, Bradley EH (2007). The association between treatment preferences and trajectories of care at the end-of-life. J Gen Intern Med.

[38] Schiff R, Sacares P, Snook J (2006). Living wills and the Mental Capacity Act: a postal questionnaire survey of UK geriatricians. Age Ageing.

[39] Bern-Klug M (2009). A framework for categorizing social interactions related to end-of-life care in nursing homes. Gerontologist.

